# *Cryptosporidium andersoni–*associated proliferative abomasitis in a roan antelope

**DOI:** 10.1177/10406387241283191

**Published:** 2024-09-25

**Authors:** Sai Fingerhood, Justine Shotton, Cecilia Gola, Martha Betson

**Affiliations:** Veterinary Pathology Centre, University of Surrey, Guildford, United Kingdom; Department of Comparative Biomedical Sciences, School of Veterinary Medicine, Faculty of Health and Medical Sciences, University of Surrey, Guildford, United Kingdom; Veterinary Department, Marwell Wildlife Zoological Park, Thompson’s Lane, Hampshire, United Kingdom; Veterinary Pathology Centre, University of Surrey, Guildford, United Kingdom; Department of Comparative Biomedical Sciences, School of Veterinary Medicine, Faculty of Health and Medical Sciences, University of Surrey, Guildford, United Kingdom; Department of Comparative Biomedical Sciences, School of Veterinary Medicine, Faculty of Health and Medical Sciences, University of Surrey, Guildford, United Kingdom

**Keywords:** *Cryptosporidium andersoni*, diarrhea, proliferative abomasitis, roan antelope

## Abstract

A 2-y-old, intact male roan antelope (*Hippotragus equinus*) was submitted for routine postmortem investigation after a prolonged history of diarrhea and weight loss. The abomasal mucosa was diffusely thickened and corrugated. Abomasal gland hyperplasia was associated with abundant apical organisms consistent with *Cryptosporidium* spp. Genomic DNA was extracted from abomasal and intestinal contents and subjected to PCR using primers specific for the 18S rRNA gene of *Cryptosporidium* spp., followed by Sanger sequencing. The sequence was >99% homologous to *Cryptosporidium andersoni. C. andersoni*–associated proliferative abomasitis has not been reported previously in a captive hippotraginid, to our knowledge.

*Cryptosporidium* spp. are zoonotic, intracellular, apicomplexan parasites; infections are an important cause of potentially life-threatening diarrhea in a broad variety of vertebrate species worldwide.^6,7^ Although many cryptosporidia have a tropism for small intestinal enterocytes, *C. andersoni*,^6^ in addition to *C. muris* and *C. serpentis*,^2^ infects abomasal and gastric epithelia. Abomasal and gastric infections with *C. andersoni* have mainly been described in cattle and camels, with occasional reports in humans.^4^ It is possible that the prevalence of *C. andersoni* infections is underestimated, even within these species, given the frequent lack of overt clinical disease.^8^

In veterinary species other than cattle, clinical findings, as well as the gross and histologic lesions associated with *C. andersoni*, have not been documented, to our knowledge. Here we describe the clinical history, and gross and histologic pathology, of a roan antelope (*Hippotragus equinus*) with *C. andersoni–*associated proliferative abomasitis.

A 2-y-old, intact male, zoo-housed, roan antelope was euthanized and submitted for postmortem examination after 5 mo of diarrhea and weight loss. The animal was co-housed with 2 other adult (7- and 12-y-old) female roan antelopes in stalls and paddocks. Premortem testing included pre- and post-treatment fecal egg counts (McMaster test; in house), CBC and biochemistry (Axiom Laboratory), fecal culture (Finn Pathologists), bovine viral diarrhea virus PCR (Axiom), *Mycobacterium avium* spp. *paratuberculosis* PCR and serology (Axiom), and bovine coronavirus serology (Axiom).

Fecal egg counts were initially low (475 eggs/g) with a reduction to no identifiable fecal parasites 2 mo after treatment with injectable ivermectin (0.2 mg/kg). Both neutrophilia (5.6 × 10^9^/L; RI: 0.6–4.0 × 10^9^/L) and hemoconcentration (hematocrit: 0.45 L/L; RI: 0.24–0.40 L/L) were mild, based on the testing laboratory’s established RIs for cattle. All PCR testing and serology were negative. The fecal culture grew an *Aeromonas* sp., which was interpreted as potentially contributory and treated with oral trimethoprim–sulfamethoxazole (TMPS). Clinically the diarrhea was unresponsive to the anthelmintics (ivermectin), TMPS, and steroids (dexamethasone IM), the latter of which was only administered within the 1.5 mo before euthanasia.

Gross postmortem examination confirmed a poor body condition with reduced adipose stores and skeletal muscle mass. Congested, prominent blood vessels imparted a cerebriform pattern to the surface of the abomasum (Fig. 1A, inset). The abomasal mucosa was diffusely thickened (up to 3 mm) with a notably corrugated surface, consistent with proliferative abomasitis (Fig. 1A). Watery fecal contents were present in the spiral and descending colons, and the mesenteric lymph nodes were firm and mildly enlarged. The rumen content pH was 5.5. No additional significant macroscopic findings were noted.

**Figure 1.**
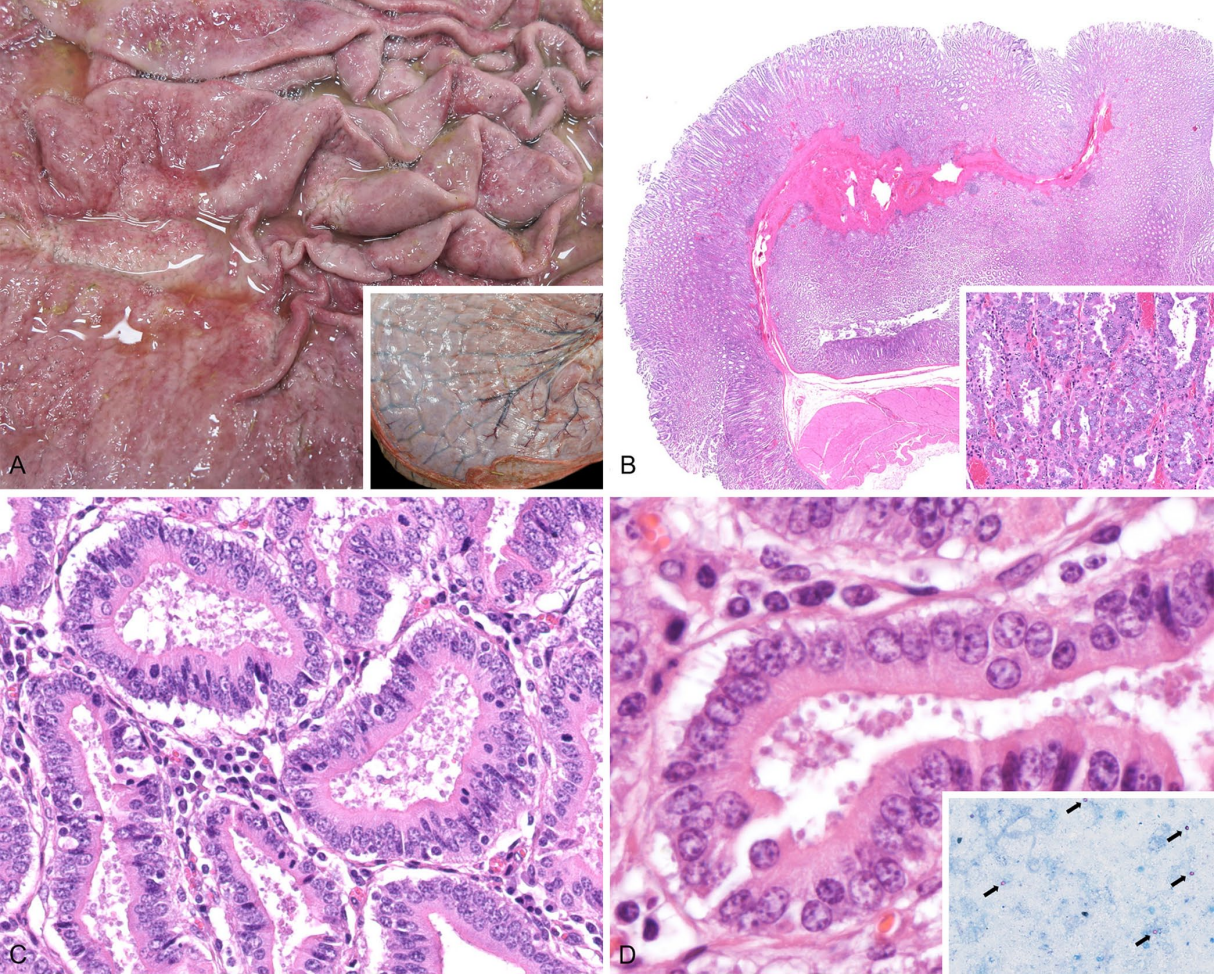
Abomasal cryptosporidiosis (*Cryptosporidium andersoni*) in a roan antelope. **A.** The abomasal mucosal folds are diffusely thickened. The mucosa itself has a cobblestoned texture. Inset: the serosa of the abomasum is swollen and glistening with prominent, engorged blood vessels bridging the surface. **B.** Abomasal fold with diffuse mucosal thickening. Abomasal glands are densely packed with elongation of the foveolae, neck, and body. H&E. Inset: hyperplastic glands include a mixture of mucous cells and parietal cells, the latter of which have prominent, eosinophilic granular cytoplasm. H&E. **C.** Abundant 6–8-μm apicomplexan organisms in apical epithelia and glands. Lymphocytes and plasma cells multifocally expand the interstitium. H&E. **D.** Higher magnification of the abomasal glands and apical apicomplexan organisms. H&E. Inset: abomasal contents smear with 6–8-μm, acid-fast organisms (arrows) that are consistent with *Cryptosporidium* spp. oocysts. Modified Ziehl–Neelsen.

Histologically, abomasal glands were diffusely elongated (Fig. 1B); the cells lining the abomasal glands were densely packed, and occasionally jumbled. The proliferative cell population was mixed, mainly consisting of surface mucous cells and parietal cells, the latter of which had prominent, eosinophilic, granular cytoplasm (Fig. 1B). On the apical surface of the abomasal epithelium and clustered at the center of glands were numerous 6–8-μm, lightly basophilic, round structures (Fig. 1C, 1D). Ziehl–Neelsen (ZN) staining of the formalin-fixed, paraffin-embedded sections did not highlight any acid-fast organisms, although ZN staining of abomasal content smears had highlighted abundant 6–8-μm, acid-fast structures (Fig. 1D, inset). The gastric lamina propria was multifocally mildly to moderately expanded with lymphocytes and plasma cells.

Additional microscopically examined sections of the alimentary tract included rumen, jejunum, colon, and ileocolic-cecal junction. All examined sections of the alimentary tract, from the rumen aborad, including the abomasum, contained moderate numbers of intraluminal, free-floating, ciliated protozoa, which were ~40 × 60 μm, ovoid, and characterized by a large paracentral macronucleus (consistent with *Balantidium* sp.). The ruminal mucosa contained occasional, small, intracorneal pustules. Lamina proprial lymphocytes and plasma cells were very mildly increased within the small intestine, cecum, and colon. Within the ileum, cecum, and colon, the lamina proprial inflammation additionally included very small numbers of neutrophils.

Genomic DNA was extracted from abomasal and intestinal contents and subjected to PCR using primers specific for the 18S rRNA gene of *Cryptosporidium* spp. using methods described previously.^9^ Extraction was followed by Sanger sequencing. All samples tested were positive by 18S PCR, and sequences were 99.8% homologous to *Cryptosporidium andersoni* (GenBank KJ531688.1, DQ448631.2). Additionally, a fecal egg count (McMaster test) was performed, and no nematode eggs or coccidial oocysts were identified.

The gross and histologic lesions associated with *C. andersoni* infection have not been reported previously in a roan antelope, to our knowledge. Searches of Google, PubMed, Web of Science, CABI Direct, and Scopus using the terms “roan antelope” and “*Cryptosporidium andersoni*” retrieved no cases. Gross and histologic morphologic features of *C. andersoni* infections have been well documented in cattle; notable histologic similarities include the association of the organisms with abomasal pit elongation, epithelial hyperplasia, and lamina proprial infiltration by lymphocytes and plasma cells.^6^ Bovine cases of *C. andersoni* have been documented most frequently as chronic infections of juvenile-to-adult animals, but have not been linked definitively to overt clinical disease nor consistent gross lesions, although an association with reduction in milk production in dairy cattle has been reported.^1,5^ This lack of clinical disease is in contrast to our case, in which loss of body condition and intractable diarrhea were sufficiently significant to be the impetus for euthanasia, and thickening of the abomasal mucosa was grossly apparent. Given that this animal was treated with steroids in the later stages of disease, it is possible that some degree of immunosuppression may have influenced the progression of this infection.

The diarrhea in our case was likely multifactorial and is partly attributed to the abomasal changes. Speculatively, the pathogenesis may include a combination of maldigestion and increased permeability of the abomasal barrier. In cattle, *C. andersoni* clinicopathologically has been associated with elevated abomasal pH (4.5–5), and elevated plasma pepsinogen, similar to ostertagiasis.^1^ If similar pH changes occur in hippotraginids, decreased active pepsin and gastrin could lead to impaired digestive function of the abomasum along with increased pH, allowing for bacterial overgrowth. This in turn could result in dysbiosis and the passage of undigested feed into the lower intestinal tract, which could result in osmotic diarrhea. Additionally, compromised intercellular junctions in the hyperplastic and inflamed mucosa may lead to the movement of serum proteins into the lumen,^3^ also potentially contributing to the development of osmotic diarrhea.

Additional potential factors contributing to the intractable diarrhea include the pustular rumenitis and the mild enterotyphlocolitis. The abundant ciliates present throughout the digestive tract were not associated with clear lesions and were likely not pathogenic in themselves. However, their presence may reflect a more systemic dysbiosis of the digestive microbiome. The mild pustular rumenitis was consistent with a chemical rumenitis, which was also supported by the mildly decreased rumen pH (5.5). This was potentially secondary to dietary factors, although neither of the other adult roan antelopes eating the same diet had clinical signs of alimentary disease. The enterotyphlocolitis was mild and nonspecific; the *Aeromonas* sp. identified with the premortem fecal culture could potentially have played a role in stimulating the inflammation; however, this is speculative, and additional testing to identify bacteria within the inflamed segments (e.g., fluorescent in situ hybridization) would be needed to assess for a pathogenic role of the bacteria.

Although rare reports of unspeciated cryptosporidia in captive hippotraginids have been documented, the susceptibility of these animals to clinical disease remains unclear.^10^ Further investigation of the prevalence of infection with *C. andersoni* in roan antelope and its association with clinical disease is warranted. Based on our findings, zookeepers and veterinarians should consider cryptosporidiosis as a differential diagnosis in cases of chronic wasting and diarrhea in this species. Cryptosporidia are zoonotic pathogens, and early diagnosis of animals in captivity may help to prevent human cases, especially in immunocompromised patients.
